# Evaluation of pathogenetic mutations in breast cancer predisposition genes in population-based studies conducted among Chinese women

**DOI:** 10.1007/s10549-020-05643-0

**Published:** 2020-04-21

**Authors:** Chenjie Zeng, Xingyi Guo, Wanqing Wen, Jiajun Shi, Jirong Long, Qiuyin Cai, Xiao-Ou Shu, Yongbin Xiang, Wei Zheng

**Affiliations:** 1 Division of Epidemiology, Department of Medicine, Vanderbilt Epidemiology Center, Vanderbilt-Ingram Cancer Center, Vanderbilt University School of Medicine, Nashville, TN USA; 2 State Key Laboratory of Oncogene and Related Genes &, Department of Epidemiology, Shanghai Cancer Institute, Renji Hospital, Shanghai Jiaotong University School of Medicine, Shanghai, China; 3 Division of Epidemiology, Department of Medicine, Vanderbilt Epidemiology Center, Vanderbilt-Ingram Cancer Center, Vanderbilt University Medical Center, 2525 West End Ave, Suite 800, Nashville, TN 37203 USA

**Keywords:** Clinical genetic testing, Breast cancer risk, Chinese women, Hereditary breast cancer syndromes

## Abstract

**Purpose:**

Limited studies have been conducted to evaluate pathogenetic mutations in breast cancer predisposition genes among Chinese women. To fully characterize germline mutations of these genes in this population, we used the whole-exome sequencing data in a population-based case–control study conducted in Shanghai, China.

**Methods:**

We evaluated exonic, splicing, and copy number variants in 11 established and 14 candidate breast cancer predisposition genes in 831 invasive breast cancer cases and 839 controls. We identified 55 pathogenic variants, including 15 newly identified in this study.

**Results:**

Approximately 8% of the cases and 0.6% of the cancer-free controls carried these pathogenetic variants (*P* = 3.05 × 10^−15^). Among cases, 3.7% had a *BRCA*2 pathogenic variant and 1.6% had a *BRCA1* pathogenic variant, while 2.5% had a pathogenic variant in other genes including *ATM, CHEK2, NBN, NF1, CDH1, PALB2, PTEN, TP53* as well as *BARD1, BRIP,* and *RAD51D*. Patients with *BRCA*1/2 pathogenic variants were more likely to have a family history of breast cancer and hormone receptor negative tumors compared with patients without pathogenic variants.

**Conclusions:**

This study highlighted the importance of hereditary breast cancer genes in the breast cancer etiology in this understudied population. Together with previous studies in East Asian women, this study suggested a relatively more prominent role of *BRCA2* compared to *BRCA1*. This study also provides additional evidence to design cost-efficient genetic testing among Chinese women for risk assessment and early detection of breast cancer.

**Electronic supplementary material:**

The online version of this article (10.1007/s10549-020-05643-0) contains supplementary material, which is available to authorized users.

## Introduction

Breast cancer is the most commonly diagnosed cancer and the leading cause of cancer deaths in Chinese women [1]. Approximately 170,000 new invasive breast cancer patients are diagnosed and 45,000 patients die of this cancer in China each year [1]. The average age at breast cancer diagnosis in among Chinese women is 45–55 years, which is approximately 10–15 years younger than that in the United States. Additionally, our previous study and other studies in Chinese patients found a higher proportion of hormone receptor-negative tumors compared with those in the United States [2, 3]. These together suggested differences in the distributions of risk factors between Chinese and their American counterparts.

Breast cancer risk is strongly influenced by genetic factors. To date, multiple breast cancer predisposition genes have been identified, mainly from studies of women of European ancestry [4–9], including *BRCA1, BRCA2, PALB2*, *CHEK2,* and *ATM*, which together accounted for 25% of the familial risk [5–9]. In addition, variants in the genes *TP53, CDH1*, *PTEN*, *STK11,* and *NF1* that cause multiple types of cancers also documented in some breast cancer patients [4]. It is clinically important to identify patients carrying pathogenic variants of breast cancer predisposition genes as it informs breast cancer risk management strategies in patients [10] and enables cascade genetic testing to identify high-risk family members. It may also inform treatment decisions. Recent studies found that BRCA status predicted response to platinum-based chemotherapy [11] and poly (ADP-ribose) polymerase inhibitors [12, 13]. The selection criteria for genetic testing in patients are in general well defined in the clinic guidelines used in the United States and other developed countries. For example, the National Comprehensive Cancer Network guideline suggested that the selection be based on age of onset, family history of relevant malignancies, hormone receptor and human epidermal growth factor receptor 2 (HER2) status, and race/ethnical groups [14]. A number of methods predicting probabilities of carrying *BRCA1/2* pathogenic variants in patients were also developed [15–18]. Currently, guidelines for genetic testing in breast cancer patients have not been established in China. Developing new guidelines or adapting existing guidelines that were developed in other populations to the Chinese population requires sufficient knowledge on the prevalence of pathogenic variants in breast cancer predisposition genes and predictors of these variants in this population. However, such information is limited, particularly for genes other than *BRCA1/2*.

To better understand the impact of pathogenic variants in breast cancer predisposition genes on breast cancer risk and their association with clinical factors, we used whole-exome sequencing data obtained from participants included in a population-based case–control study conducted in Shanghai, China to detect germline variants including single-nucleotide polymorphisms (SNPs), insertions and deletions (indels), and copy number variants (CNVs) in 11 established and 14 candidate breast cancer predisposition genes. We aimed to describe the variant spectrum of breast cancer predisposition genes and estimate the percentage of pathogenic variant carriers among cases and controls and to determine clinical factors that are associated with pathogenic variants in patients in this population.

## Materials and methods

### Study populations

This study comprised 839 invasive breast cancer cases and 839 cancer-free controls. Both cases and controls came from population-based studies conducted in urban Shanghai, China, including the Shanghai Breast Cancer Studies (SBCS-I and SBCS-II) and the Shanghai Women's Health Study (SWHS). The details of these studies have been previously described [19–22]. Briefly, for the SBCS-I, which was a case–control study, participants were recruited between 1996 and 1998. Breast cancer patients were ascertained through a rapid case-ascertainment system and the population-based Shanghai Cancer Registry. Controls were randomly selected from the general population using the Shanghai Resident Registry, a population registry containing demographic information for all residents of urban Shanghai. The inclusion criteria for controls were identical to those for cases, with the exception of a breast cancer diagnosis. Using a protocol similar to that of the SBCS-I, the SBCS-II recruited incident breast cancer cases and community controls between 2002 and 2005. The SWHS study was a population-based cohort study conducted in Shanghai with baseline surveys conducted from 1996 to 2000. Breast cancer cases were ascertained through a combination of record linkage with data from the Shanghai Cancer Registry or home visits conducted every 2 to 4 years. Medical charts and pathology slides from diagnostic hospitals were also reviewed to further verify the cancer diagnosis. The protocols for these three studies were approved by their relevant institutional review boards, and all participants provided written informed consent.

For the SBCS-I and SBCS-II, we selected cases from those who were diagnosed with breast cancer at age 58 years or younger. We also included all bilateral breast cancer cases at diagnosis (*n* = 13). Controls were selected from those without a first-degree family history of breast cancer or ovarian cancer. The age at diagnosis of cases ranged from 28 to 58 years old and the age at interview for controls ranged from 29 to 65 years old. A total of 570 cases and 570 controls were selected for the SBCS. For the SWHS, which is a prospective cohort, we selected cases with age at diagnosis not older than 55 years. We selected controls that were on average 5 years older than cases at the time of last follow-up to reduce the possibility of including mutation carriers in the group. The age at diagnosis of cases ranged from 40 to 55 years old and the age at the last follow-up for controls ranged from 42 to 70 years old. A total of 269 cases and 269 controls were selected from the SWHS.

### Whole-exome sequencing and quality control

Whole-exome sequencing of study participants was conducted using Illumina GAII sequencing platforms. The median sequence read depth was − 50X. The processing of the sequencing data has been previously described [23–25]. Briefly, paired-end reads were aligned to the human genome reference hg19 using BWA (version 0.75). Base quality recalibration and variant calling were performed using the Picard and the GATK (version 3.8) tools according to the GATK best practice guideline. Copy number variants were predicted according to our in-house pipeline [26, 27]. Briefly, we called CNV using tool including xHMM [28] and CoNIFER [29], and combined results from both tools. We removed variants with low depth of coverage (average read depth < 8) and high rate of missingness (> 2%). We conducted principal components analyses (PCAs) to remove genetic outliers using EIGINSTART based on approximately 1000 ancestry information markers as previously described [27]. We also estimated pair-wise proportion of identity by-descent (IBD) to exclude genetically identical samples, unexpected duplicate samples, and close relatives from the study. After removing 8 samples that did not pass the QC criteria, a total of 831 cases and 839 controls remained for analyses.

Based on the clinical criteria of the breast cancer diagnosis and treatment guidelines and specifications by the Chinese Cancer Society (V2015) and several previous studies, we defined patients at a high familial risk meeting any of the following criteria: (1) age at diagnosis < 35 years old; (2) bilateral breast cancer and age at diagnosis < 50 years old; (3) first-degree family history of breast or ovarian cancer.

### Functional annotation and classification of variants

Functional annotations of the identified variants were performed relative to the full set of Refseq genes, as obtained from the UCSC Genome Browser (March 2019). Genes evaluated in this study included 11 established breast cancer genes (*BRCA1, BRCA2, TP53, CDH1, PTEN, STK11, NF1, PALB2, CHEK2, ATM,* and *NBN*) and 14 candidate breast cancer genes (*ATK1, BARD1, BRIP1, CHEK1, FAM175A, FANCM, GEN1, MRE11A, RAD51B, RAD51C, RAD51D, RECQL, RINT1,* and *XRCC2*). We identified all exonic, splicing, and copy number variants in these genes. We evaluated these variants in large genomic databases including the1000 Genomes and the The Genome Aggregation Database (gnomAD) projects (version 2.1.1) if reported. Common variants identified in this study (minor allele frequency (MAF) > 1%) or reported in any public genomic database in any population with a MAF > 1% were excluded. We defined known pathogenic variants according to the following criteria: 1) pathogenic or likely pathogenic (P/LP) variants with ClinVar (last accessed in March 2019) star 2 + (i.e., multiple submitters with assertion criteria and no conflicts, expert panel or practice guideline); 2) pathogenic variants in the BRCA exchange databases (version 23, March 2019); 3) pathogenic or likely pathogenic in breast cancer by at least two submitters according to the LOVD database (version 3.0, last accessed in March 2019). We searched literature on association analyses in family or population-based studies and experimental studies on functional impact of variants in genes of interest. Additionally, in silico analyses using algorithms including CADD [30], DANN [31], SIFT [32], PolyPhen2 [33], Variant Taster [34], Variant assessor [35, 36], FATHMM-MKL [37], GERP +  + [38], PhyloP [39], and SiPhy [40] were performed. All the annotations were performed using ANNOVAR [41, 42]. According to the ACMG guideline [43, 44], based on a scoring system including minor allele frequencies across different populations, prior reports of disease association/pathogenicity, experimental studies, and number of carriers in cases and controls, we classified variants into three categories: pathogenic variants (including pathogenic and likely pathogenic variants according to the guideline), benign (benign and likely benign), and variants of unknown significance (VUS).

### Statistical analysis

We calculated the percentage of pathogenic variant carriers in cases and controls. We combined the risk alleles of pathogenic variants each gene and performed a burden test evaluating association of a mutated gene of interest with breast cancer risk. We estimated 2-sided *P* values using the Fisher’s exact test.

## Results

Demographic characteristics of cases and controls and clinical characteristics of cases are presented in Table 1. The mean age at diagnosis of cases was 46.3 years old (standard deviation (SD) = 5.2 years), and the mean age at reference of controls was 48.9 years old (SD = 7.9 years). Eight percent of patients and 2% of controls reported a family history of breast cancer. Approximately 25% of the patients had hormone receptor-negative tumors.

**Table 1:** Demographic and clinical characteristics of study participants in the Shanghai Breast Cancer Genetic Study

Characteristics	Cases	Controls
Number of participants	831	839
Age		
Mean (SD)	46.3 (5.2)	48.9 (7.9)
< 35	17 (2%)	15 (2%)
35–44	339 (41%)	309 (37%)
45–54	448 (54%)	303 (36%)
55–	27 (3%)	212 (3%)
Family history of breast cancer*		
Yes	70 (8%)	16 (2%)
No	761 (92%)	823 (98%)
Family history of any cancer		
Yes	555 (68%)	501 (60%)
No	175 (21%)	235 (25%)
Molecular subtypes		
ER + /PR +	322 (39%)	NA
ER + /PR− or ER-/PR +	131 (16%)	NA
ER-/PR-	188 (23%)	NA
ER-/PR-/Her 2 +	21 (3%)	NA
ER-/PR-/Her 2-	49 (6%)	NA

*SD* standard deviation; *ER* estrogen receptor; *PR* progesterone receptor; *HER2* human epidermal growth factor receptor 2

^*^Among first-degree relatives

**Fig. 1:**
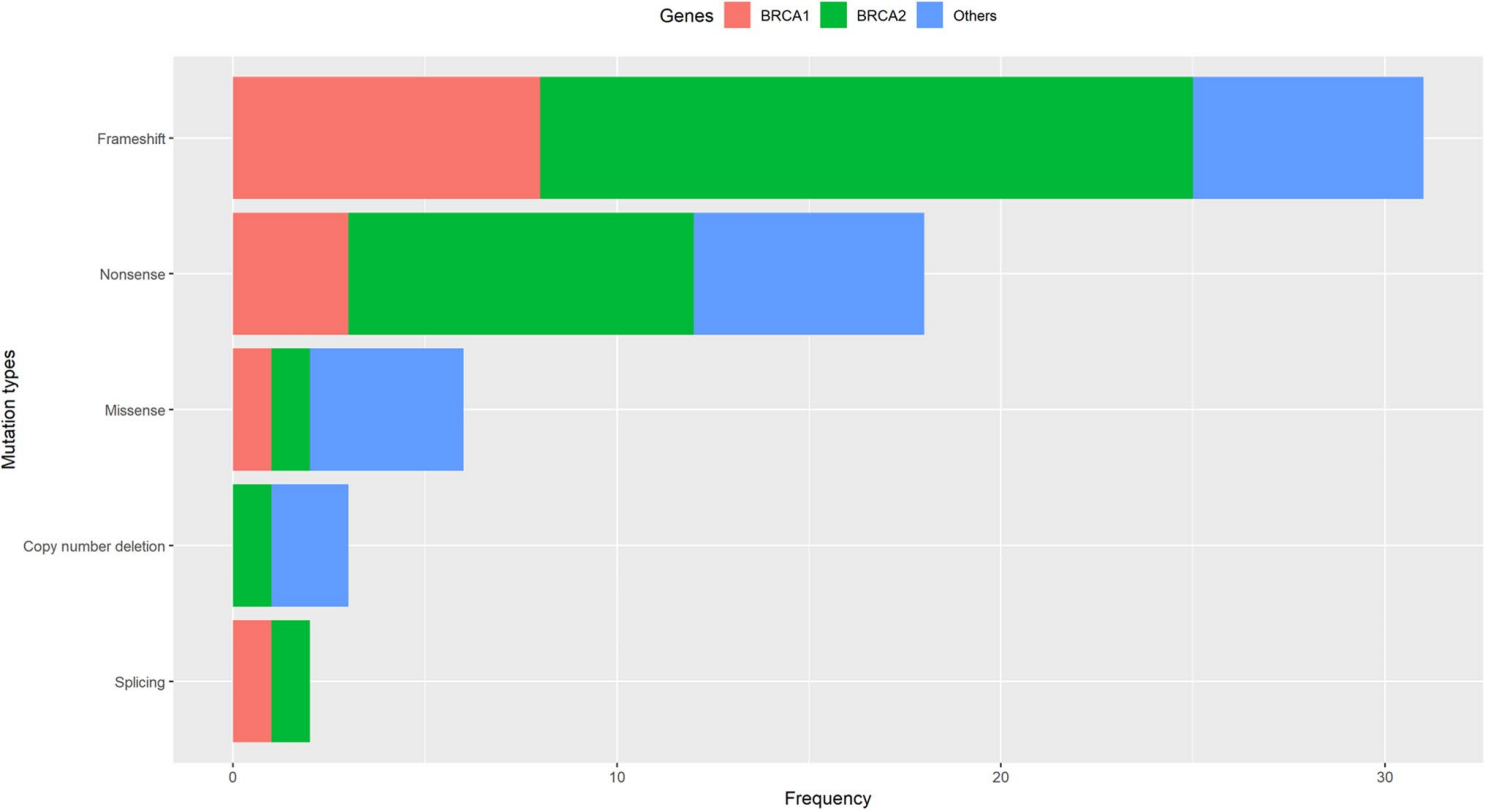
Distribution of types of pathogenic mutations in the Shanghai Breast Cancer Genetics Study

We identified 767 rare exonic, splicing variants, and large deletions in the 25 genes of interest. Among these variants, we identified 55 pathogenic variants in established breast cancer genes including *ATM*, *BRCA1*, *BRCA2*, *CDH1*, *CHEK2*, *NBN, NF1, PALB2, PTEN,* and *TP53* and 5 pathogenic variants in candidate breast cancer genes including *BARD1*, *BRIP1,* and *RAD51D* (Supplementary Table S1), including 31 frameshift indels, 18 nonsense variants, 6 missense variants, 2 splicing variants, and 3 large deletions (Fig. 1). Fifteen of these variants were newly identified in this study. Six variants were recurrent variants, including the known founder variants in Han Chinese, *BRCA1* c.5470_5477del (*n* = 3) and *BRCA2* c.C3109T (*n* = 2). We did not identify any pathogenic variant in the remaining 12 genes including *STK11, ATK1, CHEK1, FAM175A, FANCM, GEN1, MRE11A, RAD51B, RAD51C, RECQL, RINT1,* and *XRCC2*. Additionally, we identified 291 variants of unknown significance (VUS), 212 of which were newly identified in this study.

Among 831 patients with breast cancer, 65 (7.8%) carried a pathogenic variant, while among 839 cancer-free controls, 5 (0.6%) carried a pathogenic variant (Fig. 1, Table 2, Table S2). The gene contributing most to the inherited breast cancer risk was *BRCA2* (*P* = 3 × 10^−10^), accounting for 3.7% of the patients, followed by the gene *BRCA1* that accounted for an additional 1.6% of the patients (*P* = 0.01). Furthermore, 21 patients (2.5%) had a pathogenic variant in other genes including *ATM* (*n* = 4), *CHEK2* (*n* = 2), *NF1* (*n* = 2), NBN (*n* = 1), *CDH1* (*n* = 1), *PALB2* (*n* = 1), *PTEN* (*n* = 1), and *TP53* (*n* = 1) as well as *BARD1* (*n* = 2), *BRIP1* (*n* = 2), and *RAD51D* (*n* = 4) (Table 2).

**Table 2:** Frequencies of pathogenic variants in established and candidate breast cancer genes identified in cases (*n* = 831) and controls (*N* = 839) in the Shanghai Breast Cancer Genetic Study

Breast cancer predisposition genes*	Cases		Controls		*P*
	No. of carriers	%	No. of carriers	%	
Total	65	7.8	5	0.6	3.05 × 10^−15^
Established genes	57	6.6	3	0.4	1.06 × 10^−14^
*BRCA1*	13		3		
*BRCA2*	31		0		
*PALB2*	1		0		
*PTEN*	1		0		
*TP53*	1		0		
*CDH1*	1		0		
*ATM*	4		0		
*CHEK2*	2		0		
*NBN*	1		0		
*NF1*	2		0		
Candidate genes	8	1.2	2	0.2	0.12
*BARD1*	2		0		
*BRIP1*	2		0		
*RAD51D*	4		2		

^*^No pathogenic variant was identified in genes: *STK11, ATK1, CHEK1, FAM175A, FANCM, GEN1, MRE11A, RAD51B, RAD51C, RECQL, RINT1,* and *XRCC2*

We next evaluated whether clinical factors including age at diagnosis, family history of breast cancer or any cancer, and hormone receptor status of the tumor were associated with carrying a pathogenic variant in patients (Table 3). Factors that statistically significantly associated with carrying *BRCA1* pathogenic variants included a family history of breast cancer (*P* = 0.001) and hormone receptor-negative tumors (*P* = 0.0004) including triple-negative diseases (*P* = 0.01), while the only factor that was statistically significantly associated with carrying *BRCA2* pathogenic variants was a family history of breast cancer (*P* = 0.0002). No statistically significant association was found between age at diagnosis and *BRCA1* or *BRCA2* mutations. No factor was statistically significantly associated with carrying pathogenic variant in other breast cancer predisposition genes.

**Table 3:** Clinical factors associated with carrying pathogenic variants in *BRCA1/2* and other breast cancer predisposition genes in breast cancer cases (n = 831) in the Shanghai Breast Cancer Genetic Study

Characteristics	No. of patients	None^a^	*BRCA1* carriers	*BRCA2* carriers	Other carriers	*P*		
						*BRCA1* carriers vs none	*BRCA2* carriers vs none	Other carriers vs none
Age at diagnosis						0.98	0.39	0.18
Mean (SD)	–	46.3 (5.2)	46.3 (4.2)	45.3 (5.2)	47.9 (5.4)			
Range	–	28–58	39–52	33–55	34–56			
< 35	17 (2%)	15 (2%)	0 (0%)	1 (3%)	1 (5%)			
35–45	342 (41%)	317 (41%)	5 (38%)	15 (48%)	5 (23%)			
> 45	472 (56%)	434 (56%)	8 (62%)	15 (48%)	15 (71%)			
Family history of breast cancer						0.001	0.0002	0.05
Yes	70 (8%)	52 (7%)	5 (38%)	9 (29%)	4 (19%)			
No	761 (92%)	714 (93%)	8 (62%)	22 (71%)	17 (81%)			
Family history of any cancer*						1.00	0.04	0.58
Yes	555 (76%)	509 (75%)	8 (80%)	25 (93%)	13 (68%)			
No	175 (24%)	165 (25%)	2 (20%)	2 (7%)	6 (32%)			
Hormone receptor status*						0.0004	0.50	0.16
Positive	456 (71%)	428 (72%)	3 (23%)	16 (67%)	9 (56%)			
Negative	188 (29%)	163 (28%)	10 (77%)	8 (33%)	7 (43%)			
Triple-negative diseases*						0.01	0.67	0.60
Yes	49 (10%)	42 (8%)	3 (50%)	2 (11%)	2 (18%)			
No	456 (90%)	428 (92%)	3 (50%)	16 (89%)	9 (82%)			

^*^Among patients with available data only

^a^No pathogenic variants identified

## Discussion

By analyzing germline variations in breast cancer predisposition genes in population-based case–control studies using whole-exome sequencing, we found that 7.8% of cases and 0.6% of controls carried at least one pathogenic variant in 10 established and 3 candidate breast cancer predisposition genes. Specifically, among 831 cases, we found that 3.7% had a *BRCA2* pathogenic variant, 1.5% had a *BRCA1* pathogenic variant, and 2.5% had a pathogenic variant in any of the 11 other genes. Clinical factors associated with *BRCA1* pathogenic variants included a family history of breast cancer and hormone receptor status of the tumor, while for BRCA2, the only statistically significant clinical factor was a family history of breast cancer. Results from this study suggested an important role of pathogenetic variants in breast cancer risk in the general population in China. Our findings should be helpful in developing guidelines for identifying high-risk women for genetic risk evaluation of breast cancer.

Although the percentage of overall pathogenic variant carriers in our study was in general comparable to studies conducted in women of European or African ancestry [45], there were remarkable differences in contributions to breast cancer risk by each breast cancer predisposition gene among populations. For example, in our study, the gene contributing most to the breast cancer risk was *BRCA2* while in studies of European ancestry or African ancestry, it was *BRCA*1. This discrepancy has been observed in previous studies in both selected and unselected populations in China [46, 47] and in other East Asian countries including Japan and Korea [48, 49]. These indicated differences in the distribution of genetic risk factors across racial groups.

In our study, more than 2% of the patients carried pathogenic variants in genes other than *BRCA*1/2, supporting the need of using multigene panel testing in breast cancer patients in China. Currently, most of the recommendations for genetic testing for non-BRCA1/2 genes are based on specific cancer syndromes with well-studied clinical features, such as the Li-Fraumeni syndrome and Cowden syndrome. Without multigene panel testing, variants in these genes could have been neglected. For example, in this study, we identified one patient with a *TP53* pathogenic variant, whom could have been missed according to the current guidelines for genetic testing in China.

We also identified 291 VUS in nearly one-third of cases in this study, which were comparable to other previous studies. Some of these VUS are likely to be reclassified as pathogenic variants with more data in the future [50]. Given the clinical implications of genetic testing, accurate variant interpretation of VUS are critical for personalized management of cancer patients and informing cascade genetic testing among family members.

Our study has several strengths. First, the population-based study design was less susceptible to ascertainment biases that were commonly found in clinics-based studies that recruited high-risk patients. Second, whole-exome sequencing enabled us to detect large CNVs that were not routinely detected in target sequencing that is commonly used in clinical setting.

The limitations of this study included incomplete data on breast cancer subtypes. Nearly three-quarter of patients did not have data on HER2 status and nearly one-quarter did not have data on hormone receptor status. Therefore, we were not able to fully evaluate associations of breast cancer subtypes with pathogenic variants in this study. All patients included in this study were diagnosed with breast cancer at age 58 years or younger, and thus the prevalence of pathogenetic variants reported in this study is for a relatively young patient population. There were some differences in selecting controls between the SWHS and the SBCS studies. We selected older controls from the SWHS, a prospective study, to reduce the possibility of including mutation carriers in the group, while we were not able to do so in the SBCSs. However, this discrepancy did not affect our conclusion, given that 0.6% of the controls carried a pathogenic variant.

In conclusion, this is so far the largest study using whole-exome sequencing to detect the full-variant spectrum of breast cancer predisposition genes in population-based case–control studies in Chinese women. We found that pathogenic variants in breast cancer predisposition genes were highly prevalent in Chinese breast cancer patients. The gene that contributed most to inherited breast cancer risk in this population was *BRCA2,* followed by *BRCA1*, togethe*r* accounting for more than 5% of the cases*.* Patients with *BRCA1*/2 pathogenic variants were more likely to have a family history of breast cancer and hormone receptor-negative tumors. Other predisposition genes included *ATM, CHEK2, PALB2, NBN, NF1, CDH1, PTEN, TP53*, accounting for an additional 1.6% of the cases. Additional three candidate genes including *BRAD1, BRIP1*, and *RAD51D* potentially accounted for an additional 1.0% of the cases. This study provides significant data that should be useful in designing cost-efficient genetic testing of breast cancer predisposition genes for risk assessment and early detection among Chinese women.

## Electronic supplementary material

Below is the link to the electronic supplementary material.

Supplementary file1 (DOCX 18 kb)

## Data Availability

The WES data from 831 breast cancer cases and 839 controls and their clinical characterization in our study have been uploaded to the database of Genotypes and Phenotypes (dbGaP) under Sequence Read Archive (SRA) accession numbers “PRJNA560925” and “PRJNA557488” for sharing the data with the research community.
