# Supplementary material for: Evaluation of pathogenetic mutations in breast cancer predisposition genes in population-based studies conducted among Chinese women
Source: Breast Cancer Res Treat. 2020 Apr 21;181(2):465–73. doi: 10.1007/s10549-020-05643-0 (PMC7188717; doi:10.1007/s10549-020-05643-0)
Supplement: Supplementary file 1 — Supplementary file1 (DOCX 18 kb) [file 10549_2020_5643_MOESM1_ESM.docx]

**Table S1 Pathogenic variants identified in 831 breast cancer cases and 839 controls**

| **Pathogenic variants** | **Function** | **No. in cases** | **No. in controls** |
| --- | --- | --- | --- |
| **Established Breast cancer genes** |  |  |  |
| ATM:NM_000051:exon10:c.1285dupA:p.P428fs* | frameshift insertion | 1 | 0 |
| ATM:NM_000051:exon10:c.1402_1403del:p.K468fs | frameshift deletion | 2 | 0 |
| ATM:NM_000051:exon56:c.A8189C:p.Q2730P* | nonsynonymous SNV | 1 | 0 |
| BRCA1:NM_007294:exon10:c.2110_2111del:p.N704fs | frameshift deletion | 1 | 0 |
| BRCA1:NM_007294:exon10:c.3288_3289del:p.Q1096fs | frameshift deletion | 1 | 0 |
| BRCA1:NM_007294:exon10:c.3435_3436del:p.V1145fs* | frameshift deletion | 1 | 0 |
| BRCA1:NM_007294:exon10:c.3770_3771del:p.E1257fs | frameshift deletion | 1 | 1 |
| BRCA1:NM_007294:exon10:c.3916_3917del:p.L1306fs | frameshift deletion | 1 | 0 |
| BRCA1:NM_007294:exon10:c.C3268T:p.Q1090X | stopgain | 0 | 1 |
| BRCA1:NM_007294:exon10:c.G3637T:p.E1213X | stopgain | 0 | 1 |
| BRCA1:NM_007294:exon10:c.T3257A:p.L1086X | stopgain | 1 | 0 |
| BRCA1:NM_007294:exon13:c.4484+1G>T | splicing | 1 | 0 |
| BRCA1:NM_007294:exon15:c.4782delA:p.P1594fs* | frameshift deletion | 1 | 0 |
| BRCA1:NM_007294:exon17:c.G5117A:p.G1706E | nonsynonymous SNV | 1 | 0 |
| BRCA1:NM_007294:exon23:c.5470_5477del:p.I1824fs | frameshift deletion | 3 | 0 |
| BRCA1:NM_007294:exon6:c.420_421del:p.S140fs* | frameshift deletion | 1 | 0 |
| BRCA2: 13:32910401-32945237 deletion* | copy number deletion | 1 | 0 |
| BRCA2:NM_000059:exon10:c.1293_1294del:p.T431fs | frameshift deletion | 1 | 0 |
| BRCA2:NM_000059:exon10:c.1806dupA:p.G602fs | frameshift insertion | 1 | 0 |
| BRCA2:NM_000059:exon10:c.1856delA:p.Q619fs* | frameshift deletion | 1 | 0 |
| BRCA2:NM_000059:exon10:c.915_918del:p.E305fs | frameshift deletion | 1 | 0 |
| BRCA2:NM_000059:exon11:c.2257delT:p.F753fs | frameshift deletion | 1 | 0 |
| BRCA2:NM_000059:exon11:c.2440delC:p.P814fs | frameshift deletion | 1 | 0 |
| BRCA2:NM_000059:exon11:c.2806_2809del:p.K936fs | frameshift deletion | 1 | 0 |
| BRCA2:NM_000059:exon11:c.2951dupA:p.E984fs | frameshift insertion | 1 | 0 |
| BRCA2:NM_000059:exon11:c.3627_3628del:p.T1209fs | frameshift deletion | 1 | 0 |
| BRCA2:NM_000059:exon11:c.3846_3847del:p.T1282fs | frameshift deletion | 1 | 0 |
| BRCA2:NM_000059:exon11:c.5611_5615del:p.S1871fs | frameshift deletion | 1 | 0 |
| BRCA2:NM_000059:exon11:c.6288_6289del:p.P2096fs* | frameshift deletion | 1 | 0 |
| BRCA2:NM_000059:exon11:c.6331_6335del:p.K2111fs | frameshift deletion | 1 | 0 |
| BRCA2:NM_000059:exon11:c.6402_6406del:p.N2134fs | frameshift deletion | 1 | 0 |
| BRCA2:NM_000059:exon11:c.6482_6485del:p.D2161fs | frameshift deletion | 1 | 0 |
| BRCA2:NM_000059:exon11:c.6547delG:p.E2183fs* | frameshift deletion | 1 | 0 |
| BRCA2:NM_000059:exon11:c.C3109T:p.Q1037X | stopgain | 2 | 0 |
| BRCA2:NM_000059:exon11:c.C5645A:p.S1882X | stopgain | 1 | 0 |
| BRCA2:NM_000059:exon11:c.C5682G:p.Y1894X | stopgain | 1 | 0 |
| BRCA2:NM_000059:exon11:c.C5959T:p.Q1987X | stopgain | 1 | 0 |
| BRCA2:NM_000059:exon11:c.C6155A:p.S2052X | stopgain | 1 | 0 |
| BRCA2:NM_000059:exon11:c.C6155G:p.S2052X | stopgain | 1 | 0 |
| BRCA2:NM_000059:exon11:c.C6359G:p.S2120X | stopgain | 2 | 0 |
| BRCA2:NM_000059:exon18:c.C8009T:p.S2670L | nonsynonymous SNV | 1 | 0 |
| BRCA2:NM_000059:exon22:c.C8951G:p.S2984X | stopgain | 1 | 0 |
| BRCA2:NM_000059:exon25:c.C9382T:p.R3128X | stopgain | 1 | 0 |
| BRCA2:NM_000059:exon23:c.9117+2T>G* | splicing | 1 | 0 |
| BRCA2:NM_000059:exon8:c.657_658del:p.T219fs | frameshift deletion | 1 | 0 |
| CDH1:NM_004360:exon3:c.A203G:p.Y68C | nonsynonymous SNV | 1 | 0 |
| CHEK2:22:29130390-29141996* | copy number deletion | 1 | 0 |
| CHEK2:NM_007194:exon3:c.C417A:p.Y139X | stopgain | 1 | 0 |
| NBN:NM_002485:exon2:c.C127T:p.R43X | stopgain | 1 | 0 |
| NF1:NM_000267:exon25:c.3198_3199del:p.R1066fs* | frameshift deletion | 1 | 0 |
| NF1:NM_000267:exon54:c.8039_8042del:p.S2680fs* | frameshift deletion | 1 | 0 |
| PALB2:NM_024675:exon4:c.C1108T:p.Q370X | stopgain | 1 | 0 |
| PTEN:NM_000314:exon5:c.T463C:p.Y155H | nonsynonymous SNV | 1 | 0 |
| TP53:NM_000546:exon8:c.G845A:p.R282Q | nonsynonymous SNV | 1 | 0 |
| **Candidate breast cancer genes** |  |  |  |
| BARD1:2:215632205-­215674293* | copy number deletion | 1 | 0 |
| BARD1:NM_000465:exon4:c.C526T:p.Q176X | stopgain | 1 | 0 |
| BRIP1:NM_032043:exon12:c.C1741T:p.R581X | stopgain | 2 | 0 |
| RAD51D:NM_002878:exon4:c.271_272insTA:p.K91fs | frameshift insertion | 3 | 2 |
| RAD51D:NM_002878:exon8:c.C694T:p.R232X | stopgain | 1 | 0 |

*novel variants, which had not been reported in 1000 Genomes Project or Gnomad 2.1.1.

Table S2 Frequency and types of pathogenic variants among cases

| Gene | Type of pathogenic variants | Frequency |
| --- | --- | --- |
| *BRCA1* | frameshift | 8 |
| *BRCA1* | missense | 1 |
| *BRCA1* | splicing | 1 |
| *BRCA1* | nonsense | 3 |
| *BRCA2* | copy number variation | 1 |
| *BRCA2* | frameshift | 17 |
| *BRCA2* | missense | 1 |
| *BRCA2* | splicing | 1 |
| *BRCA2* | nonsense | 11 |
| other genes | copy number variation | 2 |
| other genes | frameshift | 8 |
| other genes | missense | 4 |
| other genes | nonsense | 7 |
